# In vitro activity of phospholipase A_2_ and of peptides from *Crotalus durissus terrificus* venom against amastigote and promastigote forms of *Leishmania (L.) infantum chagasi*

**DOI:** 10.1186/s40409-015-0049-0

**Published:** 2015-11-24

**Authors:** Gustavo A. C. Barros, Andreia V. Pereira, Luciana C. Barros, Airton Lourenço Jr, Sueli A. Calvi, Lucilene D. Santos, Benedito Barraviera, Rui Seabra Ferreira

**Affiliations:** Department of Tropical Diseases, Botucatu Medical School, São Paulo State University (UNESP – Univ Estadual Paulista), Botucatu, SP Brazil; Center for the Study of Venoms and Venomous Animals (CEVAP), São Paulo State University (UNESP – Univ Estadual Paulista), Botucatu, SP Brazil; CEVAP/UNESP, Caixa Postal 577, Fazenda Experimental Lageado, Rua José Barbosa de Barros, 1780, 18610-307 Botucatu, SP Brasil

**Keywords:** PLA2, Peptides, *Crotalus dutissus terrificus*, Venom, Leishmanicidal activity

## Abstract

**Background:**

American visceral leishmaniasis is caused by the intracellular parasite *Leishmania (L.) infantum chagasi*, and transmitted by the sand fly *Lutzomyia longipalpis*. Since treatment is based on classical chemotherapeutics with significant side effects, the search for new drugs remains the greatest global challenge. Thus, this in vitro study aimed to evaluate the leishmanicidal effect of *Crotalus durissus terrificus* venom fractions on promastigote and amastigote forms of *Leishmania (L.) infantum chagasi*.

**Methods:**

Phospholipase A_2_ (PLA_2_) and a pool of peptide fraction (<3 kDa) were purified from *Crotalus* venom. Furthermore, promastigotes and peritoneal macrophages of mice infected by amastigotes were exposed to serial dilutions of the PLA_2_ and peptides at intervals varying between 1.5625 μg/mL and 200 μg/mL. Both showed activity against promastigotes that varied according to the tested concentration and the time of incubation (24, 48 and 72 h).

**Results:**

MTT assay for promastigotes showed IC_50_ of 52.07 μg/mL for PLA_2_ and 16.98 μg/mL for the peptide fraction of the venom. The cytotoxicity assessment in peritoneal macrophages showed IC_50_ of 98 μg/mL and 16.98 μg/mL for PLA_2_ and peptide by MTT assay, respectively. In peritoneal macrophages infected by *Leishmania (L.) infantum chagasi* amastigotes, the PLA_2_ stimulated growth of parasites, and at higher doses reduced growth by 23 %. The peptide fraction prevented 43 % of the intracellular parasite growth at a dose of 16.98 μg/mL, demonstrating the toxicity of this dose to macrophages. Both fractions stimulated H_2_O_2_ production by macrophages but only PLA_2_ was able to stimulate NO production.

**Conclusion:**

We have demonstrated the in vitro leishmanicidal activity of the PLA_2_ and peptide fraction of *Crotalus* venom. The results encourage further studies to describe the metabolic pathways involved in cell death, as well as the prospecting of molecules with antiparasitic activity present in the peptide fraction of *Crotalus durissus terrificus* venom.

## Background

The epidemiological relevance of American visceral leishmaniasis, caused by the parasite *Leishmania (L.) infantum chagasi*, as a neglected disease has increased significantly in Latin America and especially in Brazil, where 90 % of the cases occur [1, 2]. Transmitted by the bite of *Lutzomyia longipalpis*, infected with promastigote forms, dogs are considered the principal reservoir for humans [1, 3–5].

Its treatment is carried out via chemotherapeutics such as amphotericin B and pentamidine as well as meglumine antimonate and sodium stibogluconate, all of which present high indices of serious side effects [6–8]. The infection triggers a cluster of clinical manifestations that can lead to death, if the patient is not treated. The toxicity of drugs, the difficulties of administration and the duration of treatment, allied with their low efficacy for humans and practical inefficacy for other animals, has stimulated the research of new natural leishmanicidal compounds, such as those derived from animal venoms [9].

Snake venoms are composed of a complex mixture of distinct proteins and present different pharmacological activities that can generate different active molecules [10]. The venom of *Bothrops mojeeni* can inhibit the growth of *Leishmania* spp. due to the hydrogen peroxide generated by the activity of the enzyme L-amino acid oxidase [11]. Venoms from *Cerastes*, *Vipera*, and *Naja* also exert an inhibitory effect on growth of *Leishmania infantum donovani* [12]. The venom of *Crotalus durissus cascavella* has three main fractions that present antileishmanial activity. The South American rattlesnake *Crotalus durissus terrificus* (Cdt) possesses several biologically active proteins in its venom, such as giroxin, crotamine, convulxin, crotoxin and peptides.

Crotoxin, the major toxic component of Cdt venom, is constituted by two subunits, an acid one without enzymatic action called crotapotin, and a basic one that presents the function of phospholipase A_2_ (PLA_2_), which possesses neurotoxic and myotoxic action [13, 14]. Evaluations of the in vitro action of PLA_2_ from snakes of the genus *Bothrops* against promastigote forms of *Leishmania* sp. have obtained encouraging results against these parasites [15–17]. Another important component of Cdt venom is its peptide fraction, which is composed of low-molecular-weight molecules. Despite previous descriptions of this peptide fraction having presented anesthetic action and natriuretic peptides, neither of these two aspects has been evaluated against *Leishmania.*

Considering that snake venoms constitute a great source of bioactive protein substances that can act on diverse physiological systems, the present work aimed to evaluate the action of PLA_2_ and of the peptide fraction from *Crotalus durissus terrificus* snake venom against *Leishmania (L.) infantum chagasi*, in in vitro infection of murine macrophages and in a culture of promastigotes.

## Methods

*Crotalus durissus terrificus* (Cdt) venom was kindly provided by the Center for the Study of Venoms and Venomous Animals, UNESP, Botucatu, Brazil. The entire procedure followed the ethical principles for animal experimentation adopted by the Brazilian College of Animal Experimentation (COBEA), and was approved by the Ethics Committee for Animal Experimentation at the Botucatu Medical School, UNESP, CEEA 890-2011.

Protein quantification was determined by the Bradford method [18] using bovine serum albumin (BSA) as the molecular standard (Sigma, USA). Electrophoresis was carried out under denatured conditions on 13 % (m/v) polyacrylamide, in order to evaluate the purity of the samples and the molecular masses of the proteins isolated [19].

The purification of PLA_2_ was performed utilizing the Äkta Explorer 100 (GE HealthCare®) by three chromatographic steps. In the first two, the chromatographic strategy adopted was molecular exclusion, utilizing the column Hiprep 26/60/Sephacryl S-100 HR (2.6 × 60 cm, GE HealthCare®). The chromatographic reading was done with a flow of 1 mL/min under absorbance of 280 nm. One gram of lyophilized Cdt venom was eluted in 50 mM of ammonium formate and 150 mM of NaCl, pH 3.5. The fraction eluted between the volumes 40 and 60 mL was collected and lyophilized, being re-chromatographed under the same described conditions. Next, the fraction eluted between the volumes 42 and 52 mL was collected and lyophilized again. The third step, namely purification, employed an ion-exchange strategy by means of the Hitrap DEAE column (0.7 × 2.5 cm, 1 mL, GE HealthCare®) previously equilibrated in a 6 M buffer of urea and 50 mM of Tris, pH 7.3. A 100 μL solution with a concentration of 2.7 μg/mL of purified protein fraction diluted in equilibrium buffer was injected at a flow rate of 0.5 mL/min and absorbance of 280 nm, under a gradient from 0 to 100 % of buffer of 6 M urea, 50 mM Tris and 1 M NaCl. After 27 min, the fraction of interest was collected [20].

PLA_2_ was identified by phospholipase activity [16] and by sequencing of amino acids from their N-terminal portion by the Edman Degradation Chemistry technique [21] using automated equipment, model PPSQ-21 (Shimadzu®), following the manufacturer’s standard instructions. SDS-PAGE was performed under reducing or non-reducing conditions using a 13.5 % polyacrylamide gel [16]. These procedures were employed to evaluate whether the protein isolated was of interest for the present study.

To obtain the pool of peptide fraction (<3 kDa), 300 mg of lyophilized Cdt venom was diluted in 2 mL of 0.1 % TFA (v/v) and homogenized. The supernatant was placed in a spin concentrator, model Viva Spin 2-3000 MW (GE HealthCare®) and centrifuged at 3000 rpm for 30 min. This process was repeated three times until the entire peptide fraction had passed through the concentrator [22].

The *L. (L.) infantum chagasi* promastigotes (MHOM/BR/1972/LD) were maintained in agar-blood biphasic medium, Novy-MacNeal-Nicolle (NNN), associated with liver infusion tryptose (LIT) medium supplemented with 10 % (v/v) bovine fetal serum (BFS), 10 mg/mL of gentamicin in a humid incubator (Biologic Oxygen Demand TE-381) at 25 °C, containing 5 % CO_2_ [23]. Subculturing was carried out every 15 days. The macrophages were obtained from the peritoneal cavity of BALB/c mice by washing with 3 mL of culture medium RPMI-1640 (Roswell Park Memorial Institute) supplemented with 10 % (v/v) BFS and were maintained at 37 °C in an atmosphere of 5 % CO_2_ [24].

To determine the 50 % inhibitory concentration (IC_50_) against promastigotes of *L. (L.) infantum chagasi* in the latest logarithmic phase of growth, the cultures were washed three times in phosphate buffer (PBS) and their concentration was adjusted to 10^6^/mL, starting from the counting performed in a Neubauer chamber. Subsequently, culture aliquots containing 5 × 10^5^ parasites were applied in 96-well plates with LIT medium supplemented with 10 % BFS. The PLA_2_ and the peptide fraction of the Cdt venom were dissolved in PBS, and serially diluted starting from an initial concentration of 200 μg/mL, with dilution factor 2, that were next added individually to cultures. Amphotericin B was employed as positive control (0.890 μg/mL) and PBS as negative control.

The in vitro susceptibility of *Leishmania (L.) infantum chagasi* promastigotes treated with PLA_2_ and the peptide fraction of Cdt venom was determined by the colorimetric method and by studying the mitochondrial oxidative activity using MTT tetrazolium salt at a concentration of 5 mg/mL, diluted in PBS, sterilized in a 0.22 μm methylcellulose membrane (Millipore®). Then, 20 μ/L per well was added, which was incubated for 4 h at 24 °C. The isolated incubated promastigotes were utilized as a viability control. The reenzymatic action was analyzed, whereas the precipitate was solubilized by the addition of 80 mL of SDS 10 % (m/v) dissolved in Milli-Q water; the optical density (OD) was determined by utilizing a plate reader (Multiskan MS, Uniscience®) at 550 nm. The definition of 100 % viability was based on the OD of the control [24].

To determine the 50 % inhibitory concentration (IC_50_) against intracellular amastigotes of *Leishmania (L.) infantum chagasi,* peritoneal macrophages were obtained from BALB/c mice, and the cellular concentration was adjusted to 8 × 10^5^ cells/mL. Next, the cell suspension was incubated on 16-well Nunc® microculture plates, (System Deslize™ Lab-Tek Chamber, USA) coupled with a histological slide, at the proportion of 4 × 10^5^ cells/well and maintained at 37 °C in an atmosphere of 5 % CO_2_ for a period of 24 h so that the macrophages would adhere to the plate. The supernatant was removed and added to RPMI-1640 medium supplemented with 10 % BFS. After this period, PLA_2_ and the peptide fraction of the Cdt venom were dissolved in PBS, and serially diluted starting from an initial concentration of 100 μg/mL, with dilution factor 2, that were next added individually to cultures. Untreated infected macrophages were used as control. The assays were performed in triplicate, and evaluated after 24, 48 and 72 h by optical microscopy. The slides were fixed with methanol and stained with Giemsa. IC_50_ was determined by counting 500 macrophages per well and evaluating the number of infected macrophages. Amphotericin B was employed as positive control (IC_50_ 0.890 μg/mL) and PBS as negative control [25].

To evaluate the cytotoxicity in mammalian cells, the peritoneal macrophages (4 × 10^5^ cells/well) were incubated with 200 μg/mL of PLA_2_ and with 100 μg/mL of peptide fraction from Cdt and serially diluted at 37 °C in an atmosphere of 5 % CO_2_ for a period of 24 h and 48 h [26, 27]. Amphotericin B (0.890 μg/mL) was employed as positive control. Mammalian cellular viability was determined by the MTT assay using the microplate reader MS Multi-skan (Uniscience®) at 550 nm.

For the determination of nitric oxide (NO) production, the infected macrophages were incubated in microtubes containing different concentrations of PLA_2_ and of the peptide fraction (200 μg/mL, 100 μg/mL, 50 μg/mL, 25 μg/mL, 12.5 μg/mL, 6.25 μg/mL, 3.125 μg/mL, 1.5625 μg/mL) for 24, 48 and 72 h. After each incubation, the recollected supernatant was applied into 96-well microplates for measurement of nitrite (NO_2_) and nitrate (NO_3_). The nitrite concentration was verified by the reaction of Griess. The results were obtained by means of MS Multiskan microplates (Uniscience®) at 540 nm, in comparison to a zero standard consisting of Griess reagent. All the measurements were performed in triplicate and the results were expressed in micromoles of NO/2 × 10^5^ cells, starting from the standard curve established by known concentrations of molarities from 0.39 to 200 μM NO_2_^-^. The control was constituted by cells treated with lipopolysaccharide (LPS), and by untreated cells [28, 29].

The production of H_2_O_2_ was evaluated by incubation of peritoneal macrophages with different concentrations of PLA_2_ and of the peptide fraction (200 μg/mL, 100 μg/mL, 50 μg/mL, 25 μg/mL, 12.5 μg/mL, 6.25 μg/mL, 3.125 μg/mL, 1.5625 μg/mL), for 24, 48 and 72 h. Added to the macrophages after each incubation period was 100 μL of a buffer containing 140 mM NaCl, 10 mM phosphate, pH 7, 5.5 mM Dextrose, 0.56 mM phenol red and 0.01 mg/mL peroxidase Type II (Sigma Chemical Co, USA) in each well. The plates were incubated at 37 °C, in an atmosphere of 5 % CO_2_ for 4 h, and the reaction was stopped for the addition of 0.01 mL of 1 N NaOH. The absorbance was determined in an automatic microplate reader (MD 5000®, Dynatech Laboratories Inc., USA) with a 620 nm filter in relation to a “zero standard” consisting of phenol red and 1 N NaOH. All measurements were performed in triplicate whereas H_2_O_2_ production was expressed in nanomoles/2 × 10^5^ cells, according to the standard curve established for each test. Under the experimental conditions, the curve was generated with the H_2_O_2_ concentrations of 0.5; 1.0; 2.0; 4.0 and 8.0 mM. The control was composed of cells treated with phorbol myresteate acetate (PMA) and of untreated cells [30].

Data (mean ± SD, MPa) were analyzed by one-way ANOVA and Tukey test (*p* < 0.05). Nonparametric data were expressed as median and were analyzed using the Mann-Whitney test (*p* < 0.05). All analysis was performed with GraphPad PRISM® Version 1.5 software (GraphPad Software Inc., USA).

## Results

### Purification of PLA_2_ and of peptide fraction from Cdt venom

**Fig. 1:**
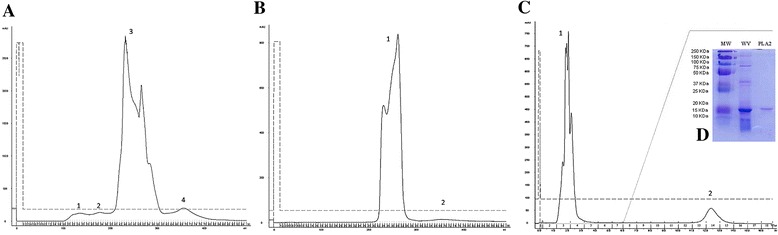
**a** Chromatographic profile of *Crotalus durissus terrificus* venom by molecular exclusion technique*.* The peaks correspond to convulxin [1], giroxin [2], crotoxin [3] and crotamine [4]. **b** Chromatographic profile of crotoxin fraction of *Crotalus durissus terrificus* venom by molecular exclusion step. **c** Chromatographic profile of crotoxin fraction of *Crotalus durissus* venom by ion exchange step, evidencing crotapotin [1] and PLA_2_ [2]. **d** SDS-PAGE of weight marker (WM), whole venom (WV) and PLA_2_

**Fig. 2:**
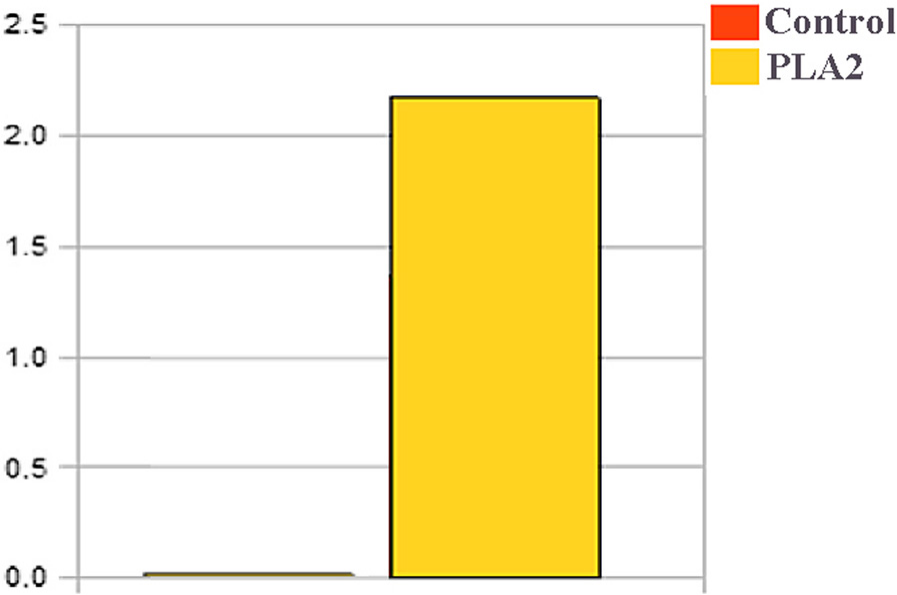
Phospholipase activity of peak 3.1.2 purified from *Crotalus durissus terrificus* venom

Figure 1 – a shows the chromatographic profile resulting from the molecular exclusion of venom from Cdt. The eluted peaks between 40 and 60 mL (peak 3) were re-chromatographed by the same technique (Fig. 1 – b). Then, the peaks eluted between 43 and 53 mL (peak 3.1) were submitted to ion-exchange chromatography. Figure 1 – c shows the elution of two distinct peaks. Peak 3.1.2 was identified as PLA_2_ and confirmed by SDS-PAGE (Fig. 1 – d), phospholipase activity (Fig. 2) and Edman sequencing (data not shown).

### Leishmanicidal activity

**Fig. 3:**
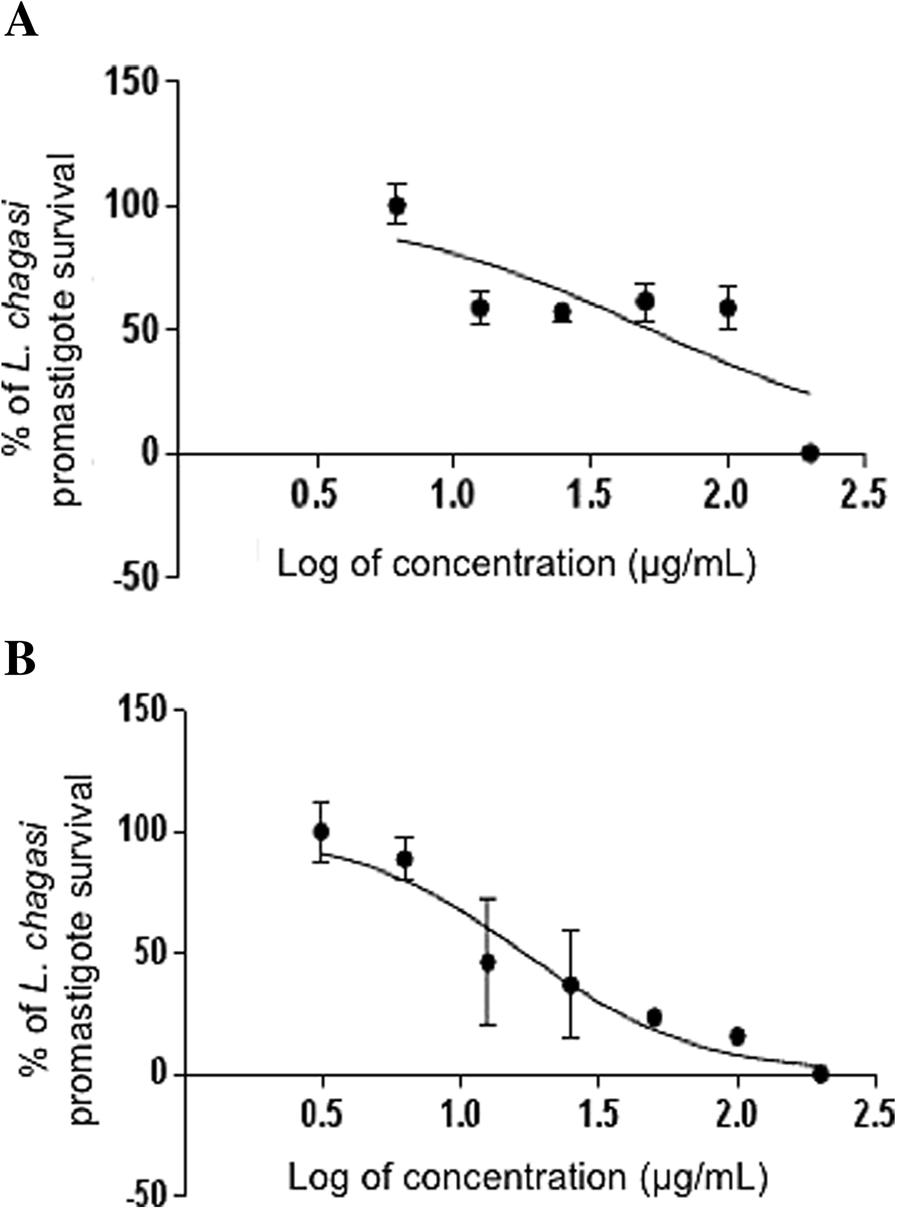
**a** Dose-response curve of MTT assay for leishimanicidal activity of PLA_2_ and (**b**) of peptide fraction from *Crotalus durissus terrificus* venom in promastigotes of *Leishmania (L.) infantum chagasi*

Figure 3 (a and b) displays leishmanicidal activities of PLA_2_ toxin and of the Cdt peptide fraction, respectively. The MTT colorimetric method in microplates revealed that the IC_50_ levels were 52.07 μg/mL for PLA_2_ and 16.98 μg/mL for the peptide fraction. Amphotericin B (positive control) showed IC_50_ 0.089 μg/mL.

### Cytotoxic activity in macrophages

**Fig. 4:**
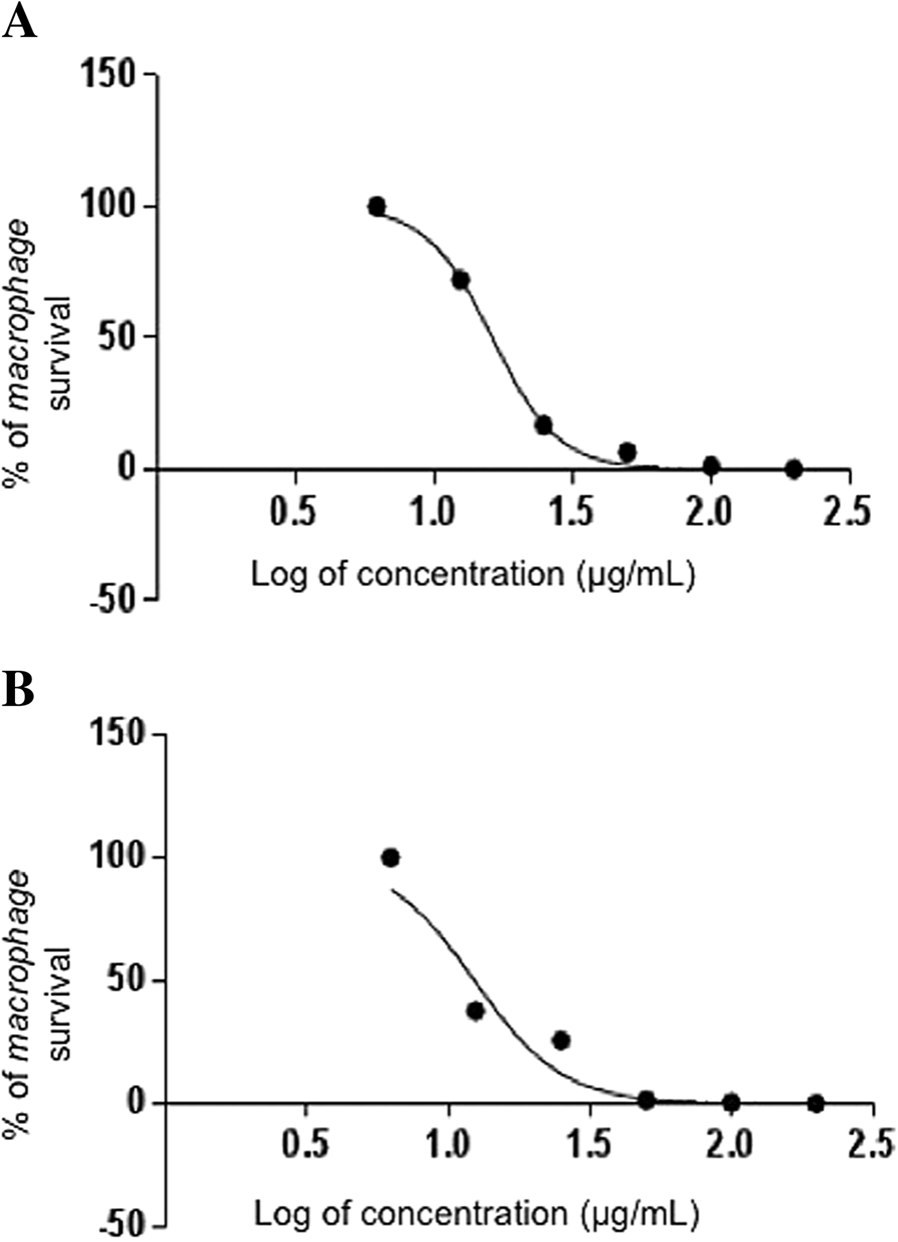
**a** Dose-response curve of MTT assay for cytotoxicity in murine macrophages of PLA_2_ and of (**b**) peptide fraction from *Crotalus durissus terrificus* venom

Figure 4 (a and b) shows the cytotoxicity of PLA_2_ and the peptide fraction of Cdt by utilizing peritoneal macrophages of BALB/c mice, as evaluated via MTT colorimetry. The IC_50_ levels were, respectively, 52.07 μg/mL for PLA_2,_ and 16.98 μg/mL for the peptide fraction.

### Determination of production of Nitric Oxide (NO) and Hydrogen Peroxide (H_2_O_2_)

**Fig. 5:**
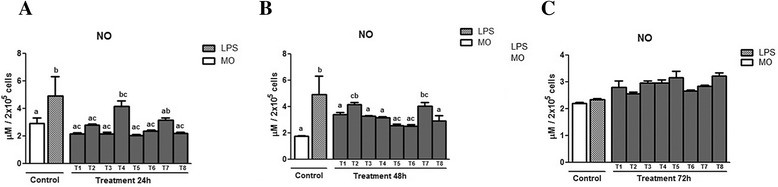
Means ± standard deviation of the mean (*p* < 0.05) of NO production in supernatant of murine macrophages infected by *L. (L.) chagasi* treated with PLA_2_ at three different incubation moments: (**a**) 24 h, (**b**) 48 h, and (**c**) 72 h. The control was done without treatment (MO) and with lipopolysaccharide (LPS). The PLA_2_ doses utilized in the treatment (T) were T1 = 100 μg/mL; T2 = 50 μg/mL; T3 = 25 μg/mL; T4 = 12.5 μg/mL; Tr5 = 6.25 μg/mL; T6 = 3.125 μg/mL; T7 = 1.5625 μg/mL; T8 = 0.78125 μg/mL

Figure 5 (a, b and c) displays the NO production of supernatant from murine macrophages infected by *L. (L) chagasi* treated with PLA_2_, measured at three different incubation moments (24, 48, and 72 h). At 24 h (Fig. 4 – a), NO production by the lipopolysaccharide-treated control (LPS) was greater than that of the control constituted by untreated macrophages (MO). In relation to the production of NO between the treatments with PLA_2_ and the controls, the treatments T1, T3, T5, T6 and T8 did not differ from the MO control. As to the LPS control, these treatments produced less NO. The treatment T4 differed from the other treatments T1, T3, T5, T6 and T8 and the MO control, but did not present a difference in relation to the LPS control. The treatment T7 differed significantly only from the MO control.

After 48 h of incubation (Fig. 5 – b), treatment T2 presented higher production than the untreated group, but did not differ from the LPS control. Treatment T7 presented greater production than the untreated control, but showed no difference in relation to the LPS-treated control. The treatments T5 and T6 produced less NO than the LPS-treated group. At 72 h (Fig. 5 – c), a tendency toward increasing NO production was found only in the PLA_2_-treated groups although there was no significant difference in relation to the controls. The peptide fraction was unable to stimulate the production of NO by macrophages.

**Fig. 6:**
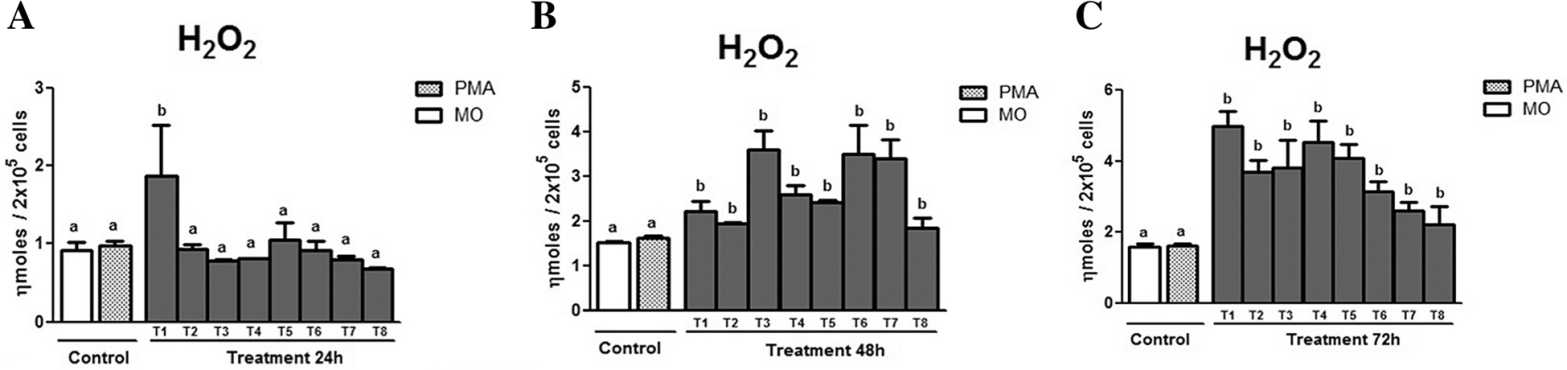
Means ± standard deviation of the mean (*p* < 0.05) of H_2_O_2_ production by peritoneal macrophages of murine macrophages infected by *L. (L.) chagasi* treated with PLA_2_ at three different incubation moments: (**a**) 24 h, (**b**) 48 h, and (**c**) 72 h. The control was performed without treatment (MO) and with stimulation by PMA. The PLA_2_ doses utilized in the treatment (T) were T1 = 100 μg/mL; T2 = 50 μg/mL; T3 = 25 μg/mL; T4 = 12.5 μg/mL; T5 = 6.25 μg/mL; T6 = 3.125 μg/mL; T7 = 1.5625 μg/mL; T8 = 0.78125 μg/mL

Figure 6 (a, b and c) shows the production of H_2_O_2_ in the supernatant of macrophages treated with PLA_2_ at 24, 48 and 72 h. At 24 h of incubation (Fig. 5 – a), a difference in relation to the two controls was observed only in treatment T1. At 48 and 72 h (Fig. 6 – b and c), an increase of H_2_O_2_ production was found, most prominently after 72 h, when all the treated groups differed (*p* < 0.05) from the controls. There was no difference among the treatments.

**Fig. 7:**
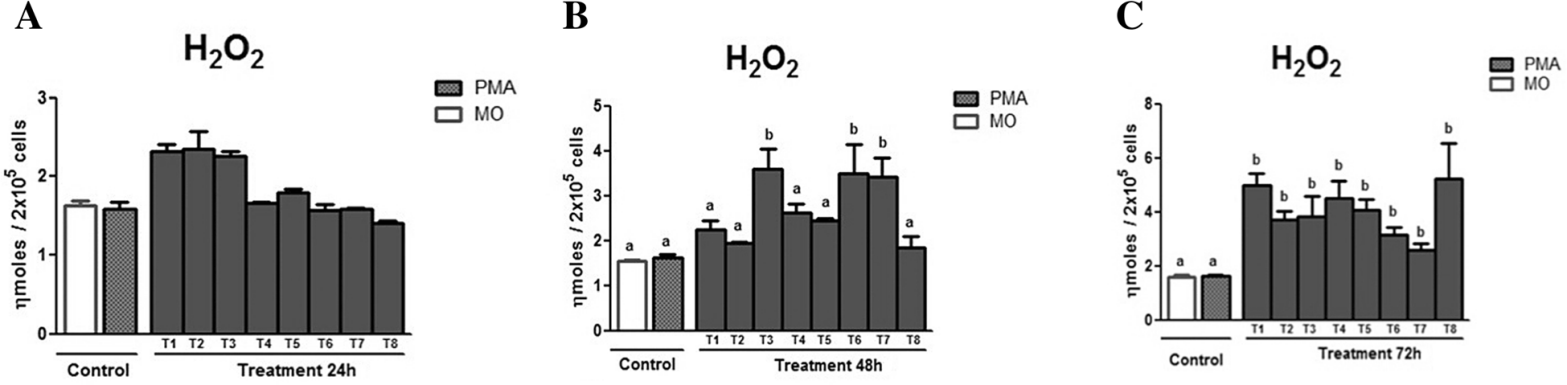
Means ± standard deviation of the mean (*p* < 0.05) of H_2_O_2_ production by peritoneal macrophages of murine macrophages infected by *L. (L.n chagasi* treated with Cdt peptide fraction, at three different incubation moments: (**a**) 24 h, (**b**) 48 h, and (**c**) 72 h. The control was done without treatment (MO) and with stimulation by PMA. The PLA_2_ doses utilized in the treatment (T) were T1 = 100 μg/mL; T2 = 50 μg/mL; T3 = 25 μg/mL; T4 = 12.5 μg/mL; T5 = 6.25 μg/mL; T6 = 3.125 μg/mL; T7 = 1.5625 μg/mL; T8 = 0.78125 μg/mL

Figure 7 (a, b and c) displays the H_2_O_2_ production in the supernatant of macrophages treated with Cdt peptide fraction at 24, 48, and 72 h. At 24 h of incubation (Fig. 7 – a), the controls did not differ from the treatments (*p* < 0.05). At 48 h of incubation (Fig. 7 – b), a significantly greater production of H_2_O_2_ was observed in treatments T3, T6 and T7 (*p* < 0.05) in relation to the controls MO and PMA. After 72 h of incubation, in all the treatments, the H_2_O_2_ production was higher (*p* < 0.05) than those of PMA and the MO controls.

## Discussion

An antiparasitic effect of snake venom has been amply described with the aim of discovering target molecules. The ultrastructural alterations caused by these venoms in parasites involve mitochondrial edema and kinetoplast dysregulation or intense cytoplasmic vacuolization and enlargement of the flagellar pocket [17, 31]. The venoms can affect the respiratory chain through ATP leakage in promastigotes and, therefore, these organelles would be the first to suffer from the treatment [11, 31].

In our present work, we have noted that the PLA_2_ isolated and purified from the venom of *Crotalus durissus terrificus* (Cdt) was able to inhibit the proliferation of *Leishmania chagasi* promastigotes at a rate from 50 % to 83 % at doses from 50 to 200 μg/mL, respectively. It was also observed that this inhibition rate was dose-dependent.

The action of PLA_2_ from venom of snakes of the genus *Bothrops* in the inhibition of *Leishmania* promastigote growth was described against *Leishmania major, L. (L.) amazonensis*, *L. (V.) braziliensis,* and L. *chagasi* [15–17]. The results found for PLA_2_ from Cdt corroborate these observations, despite small differences in the composition of PLA_2_ and in the susceptibility of the parasites. The PLA_2_ of Cdt is an enzymatically active Asp-49 whose action is associated with its catalytic site while it interacts hydrophobically with the plasma membrane penetrating into the lipid bilayer, causing an electrostatic perturbation and thereby rupturing the membrane [18, 27, 32, 33]. This direct action may have caused the inhibition of promastigote growth observed in our study.

In vivo studies have shown that promastigotes of *Leishmania amazonensis* treated with PLA_2_ purified from *Crotalus durissus colilineatus* venom augment the size of lesions in BALB/c mice. A prior study indicated that PLA_2_ appears to be a factor in the progression of cutaneous leishmaniasis, since macrophages treated with PLA_2_ presented elevated levels of prostaglandin E_2_ (PGE_2_) (an inflammatory lipid mediator) and inhibited levels of IL-2, a cytokine associated with Th-1 response [34]. The results of the present study corroborate these authors since weaker dilutions of the PLA_2_ utilized to treat the peritoneal macrophages infected by *L. (L) infantun chagasi* amastigotes induced the proliferation of parasites inside the macrophages, while at doses higher than 50 μg/mL they were able to diminish the infection by up to 27.3 %.

The low-molecular weight compounds found in snake venoms have aroused the interest of the pharmaceutical industry due to their great variety of pharmacological effects. The peptides that have already been isolated from snake venoms include: type-C natriuretic peptides, bradykinin-potentiating peptides (BPPs), sarafotoxins, waglerins and peptides that present inhibitory activity against metalloproteinases, and antimicrobial peptides [25, 35].

The antimicrobial peptides, which represent an extremely diverse group, participate primarily in the innate immune system by acting as an initial barrier of immune defense in many organisms, including plants, insects, bacteria and vertebrates [26, 36, 37]. The peptide Pep5Bj, from the venom of *Bothrops jararaca*, presents a high level of inhibitory activity against the growth of the fungi *Fusarium oxysporum* and *Colletotrichum lindemuthianum* and the yeasts *Candida albicans* and *Saccharomyces cerevisiae* [38].

The peptide fraction of Cdt venom presented an IC_50_ of 16.98 μg/mL for antileishmanial activity against the promastigotes, equivalent to the cytotoxic activity IC_50_ of 16.98 μg/mL, and is able to inhibit the growth of not only promastigotes, but also 43 % of amastigotes at the same dose.

An important factor in leishmaniases is the action of macrophages, since they are the main host cells of the parasite, and also play an important role in the control of parasites, by means of producing cytokines and metabolic oxygen compounds such as nitric oxide (NO) [37, 39].

The action mechanism of NO, as well as its specific targets within the metabolic pathways in *Leishmania* sp., were previously described as was their susceptibility to NO production by the host cell [36, 37]. In our study, the quantity of NO produced during the infection was measured at 24, 48, and 72 h, showing different results among these moments. At 24 h, the controls differed from treatment T7 (1.5625 μg/mL). After 48 h of treatment, there was a difference between the control groups, as well as between some of the treated groups. Yet at 72 h, greater NO production was shown, even in the controls.

The production of NO, one of the main microbicidal agents from murine macrophages, can be stimulated by the action of PLA_2_ from Cdt venom [40]. However, some species of *Leishmania*, such as *L. Amazonensis* and *L. chagasi*, are able to inhibit NO production [41]. In this manner, we may infer that in the first 24 h of treatment, the parasites had supported the cellular defenses through some mechanism of escape. But after 72 h the defenses produced by the macrophages would become sufficient to suppress the modulation of the parasite in the macrophage defenses. In this sense, the action of PLA_2_ impeded the parasite from maintaining its escape mechanism, a fact also observed in a prior study that measured the production of TNF-α, NO, and IL-10 in treatment with PLA_2_ from *B. pauloensis* in experimental infection of murine macrophages with *L. amazonesis* [17].

It is important to emphasize that in relation to NO production by a pool of peptides, there was no difference between the treatments and the controls, in any of the readings.

H_2_O_2_ is an antiparasitic compound that is produced by defense cells such as macrophages, when challenged by such intracellular parasites as *Leishmania* sp. A difference in H_2_O_2_ production was observed in our study only at 48 h of treatment.

Previous studies demonstrate the resistance of *Leishmania* spp. to H_2_O_2_, where it could be noted that the parasites produce catalase and superoxide dismutase, enzymes that confer an efficient escape mechanism from the action of H_2_O_2_ [15, 17, 35]. This resistance of the parasite to H_2_O_2_ production may account for the low production of this metabolite in the first 24 h, after which the activity of the peptide fraction perturbed the metabolism of the parasites, by which the parasite ceases or diminishes the production of enzymes that degrade H_2_O_2_.

## Conclusion

Due to the high toxicity presented by drugs utilized for the treatment of leishmaniases and their important side effects, it has become necessary to seek new molecules with therapeutic potential against these diseases. In this context, the present work has demonstrated in vitro leishmanicidal activity of the PLA_2_ and peptide fraction of *Crotalus dutissus terrificus* venom. New studies must be performed to describe the metabolic pathways involved in the process of cellular death. Furthermore, the prospecting of molecules whose peptide fraction presents antiparasitic activity, as well as the immunomodulation of these new molecules, must be investigated.

## References

[1] Paiva-Cavalcanti M, Regis-da-Silva CG, Gomes YM (2010). Comparison of real-time PCR and conventional PCR for detection of *Leishmania (Leishmania) infantum* infection: a mini-review. J Venom Anim Toxins Incl Trop Dis.

[2] Costa CHN (2011). How effective is dog culling in controlling zoonotic visceral leishmaniasis? A critical evaluation of the science, politics and ethics behind this public health policy. Rev Soc Bras Med Trop.

[3] Camargo LB, Langoni H (2006). Impact of leishmaniasis on public health. J Venom Anim Toxins Incl Trop Dis.

[4] OPAS Organização Panamericana de Saúde. Encuentro sobre vigilancia, prevención y control de leishmaniasis visceral (LV) en el Cono Sur de Sudamérica. http://new.paho.org/hq/index.php?option=com_docman&task=doc_view&gid=16961&Itemid=. Acessed 02 dez 2012.

[5] Dantas-Torres F, Solano-Gallego L, Baneth G, Ribeiro VM, de Paiva-Cavalcanti M, Otranto D (2012). Canine leishmaniosis in the Old and New Worlds: unveiled similarities and differences. Trends Parasitol.

[6] Berman J (1998). Chemotherapy of leishmaniasis: recent advances in the treatment of visceral disease. Curr Opin Infect Dis.

[7] Gontijo CMF, Melo MN (2004). Leishmaniose visceral no Brasil: quadro atual, desafios e perspectivas. Rev Bras Epidemiol.

[8] Ordónez-Gutiérrez L, Espada-Fernández R, Dea-Ayuela MA, Torrado JJ, Bolás-Fernandez F, Alunda JM (2007). In vitro effect of new formulations of amphotericin B on amastigote and promastigote forms of *Leishmania infantum*. Int J Antimicrob Agents.

[9] Sampaio RNR, Lucas IC, Takami HL (2007). Inefficacy of the association N-methyl glucamine and topical miltefosine in the treatment of experimental cutaneous leishmaniasis by *Leishmania* (*Leishmania*) *amazonensis*. J Venom Anim Toxins Incl Trop Dis.

[10] Costa TR, Burin SM, Menaldo DL, de Castro FA, Sampaio SV. Snake venom L-amino acid oxidases: an overview on their antitumor effects. J Venom Anim Toxins Incl Trop Dis. 2014;20(23). doi:10.1186/1678-9199-20-23. 10.1186/1678-9199-20-23 PMC4060840 24940304

[11] Tempone AG, Andrade HF, Spencer PJ, Lourenço CO, Rogero JR, Nascimento N (2001). *Bothrops moojeni* venom kills *Leishmania* spp. with hydrogen peroxide generated by its L-amino acid oxidase. Biochem Biophys Res Commun.

[12] Fernandez-Gomes R, Zerrouk H, Sebti F, Loyens M, Benslimane A, Ouaissi MA (1994). Growth inhibition of *Trypanosoma cruzi* and *Leishmania donovani infantum* by different snake venoms: preliminary identification of proteins from *Cerastes cerastes* venom which interact with the parasites. Toxicon.

[13] Rübsamen K, Breithaupt H, Habermann E (1971). Biochemistry and pharmacology of the crotoxin complex I. Subfractionation and recombination of the crotoxin complex. Naunyn Schmiedebergs Arch Pharmakol.

[14] Hendon RA, Tu AT (1979). The role of crotoxin subunits in tropical rattlesnake neurotoxic action. Biochim Biophys Acta.

[15] Stábeli RG, Amui SF, Sant’Ana CD, Pires MG, Nomizo A, Monteiro MC (2006). *Bothrops moojeni* myotoxin-II, a Lys49-phospholipase A2 homologue: an example of function versatility of snake venom proteins. Comp Biochem Physiol C Toxicol Pharmacol.

[16] Costa TR, Menaldo DL, Oliveira CZ, Santos-Filho NA, Teixeira SS, Nomizo A (2008). Myotoxic phospholipases A(2) isolated from *Bothrops brazili* snake venom and synthetic peptides derived from their C-terminal region: cytotoxic effect on microorganism and tumor cells. Peptides.

[17] Nunes DC, Figueira MM, Lopes DS, De Souza DL, Izidoro LF, Ferro EA (2013). BnSP-7 toxin, a basic phospholipase A2 from *Bothrops pauloensis* snake venom, interferes with proliferation, ultrastructure and infectivity of *Leishmania (Leishmania) amazonensis*. Parasitology.

[18] Bradford MM (1976). A rapid and sensitive method for the quantitation of microgram quantities of protein utilizing the principle of protein-dye binding. Anal Biochem.

[19] Laemmli UK (1970). Cleavage of structural proteins during the Assembly of the Head of Bacteriophage T4. Nature.

[20] Hendon RA, Fraenkel-Conrat H (1971). Biological roles of the two components of crotoxin. Proc Natl Acad Sci.

[21] Edman P, Begg G (1967). A protein sequenator. Eur J Biochem.

[22] Barreto SA, Chaguri LC, Prezoto BC, Lebrun I (2012). Characterization of two vasoactive peptides isolated from the plasma of the snake *Crotalus durissus terrificus*. Biomed Pharmacother.

[23] Diaz BL, Arm JP (2003). Prostaglandins, leukotrienes and essential fatty acids. ScienceDirect.

[24] Tempone AG, Pimenta DC, Lebrun I, Sartorelli P, Taniwaki NN, de Andrade HF (2008). Antileishmanial and antitrypanosomal activity of bufadienolides isolated from the toad *Rhinella jimi* parotoid macrogland secretion. Toxicon.

[25] Reimão JQ, Colombo FA, Pereira-Chioccola VL, Tempone AG (2012). Effectiveness of liposomal buparvaquone in an experimental hamster model of *Leishmania* (L.) *infantum chagasi*. Exp Parasitol.

[26] Páramo L, Lomonte B, Pizarro-Cerdá J, Bengoechea JA, Gorvel JP, Moreno E (1998). Bactericidal activity of Lys49 and Asp49 myotoxic phospholipases A_2_ from *Bothrops asper* snake venom – synthetic Lys49 myotoxin II-(115–129)-peptide identifies its bactericidal region. Eur J Biochem.

[27] Barbosa PS, Martins AM, Havt A, Toyama DO, Evangelista JS, Ferreira DP (2005). Renal and antibacterial effects induced by myotoxin I and II isolated from *Bothrops jararacussu* venom. Toxicon.

[28] Green LC, Wagner DA, Glogowski J, Skipper PL, Wishnok JS, Tannenbaum SR (1982). Analysis of nitrate, nitrite, and [15 N] nitrate in biological fluids. Anal Biochem.

[29] Tsukahara Y, Morisaki T, Horita Y, Torisu M, Tanaka M (1999). Phospholipase A2 mediates nitric oxide production by alveolar macrophages and acute lung injury in pancreatitis. Ann Surg.

[30] Pick E, Mizel D (1981). Rapid microassays for the measurement of superoxide and hydrogen peroxide production by macrophages in culture using an automatic enzyme immunoassay reader. J Immunol Methods.

[31] Deolindo P, Teixeira-Ferreira AS, Melo EJ, Arnholdt AC, Souza WD, Alves EW (2005). Programmed cell death in *Trypanosoma cruzi* induced by *Bothrops jararaca* venom. Mem Inst Oswaldo Cruz.

[32] Kini RM, Evans HJ (1989). A model to explain the pharmacological effects of snake venom phospholipases. Toxicon.

[33] Rufini S, de Vito P, Balestro N, Pescatori M, Luly P, Incerpi S (1999). PLA2 stimulation of Na(+)/H(+) antiport and proliferation in rat aortic smooth muscle cells. Am J Physiol.

[34] Passero LF, Tomokane TY, Corbett CE, Laurenti MD, Toyama MH (2007). Comparative studies of the anti-leishmanial activity of three *Crotalus durissus* ssp. venoms. Parasitol Res.

[35] Brogden KA (2005). Antimicrobial peptides: pore formers or metabolic inhibitors in bacteria?. Nat Rev Microbiol.

[36] Balestieri FM, Queiroz AR, Scavone C, Costa VM, Barral-Netto M, Abrahamsohn IA (2002). *Leishmania* (*L.*) *amazonensis*-induced inhibition of nitric oxide synthesis in host macrophages. Microbes Infect.

[37] Balaraman S, Tewary P, Singh VK, Madhubala R (2004). *Leishmania donovani* induces interferon regulatory factor in murine macrophages: a host defense response. Biochem Biophys Res Commun.

[38] Gomes VM, Carvalho AO, da Cunha M, Keller MN, Bloch CJR, Deolindo P (2005). Purification and characterization of a novel peptide with antifungal activity from *Bothrops jararaca* venom. Toxicon.

[39] Tripathi P, Singh V, Naik S (2007). Immune response to leishmania: paradox rather than paradigm. FEMS Immunol Med Microbiol.

[40] Sampaio SC, Rangel-Santos AC, Peres CM, Curi R, Cury Y (2005). Inhibitory effect of phospholipase A_2_ isolated from *Crotalus durissus terrificus* venom on macrophage function. Toxicon.

[41] Santos PL, Costa RV, Braz JM, Santos LF, Batista AC, Vasconcelos CR (2012). *Leishmania chagasi* naturally resistant to nitric oxide isolated from humans and dogs with visceral leishmaniasis in Brazil. Nitric Oxide.

